# Modeling of *In-Utero* and *Intra-Partum* Transmissions to Evaluate the Efficacy of Interventions for the Prevention of Perinatal HIV

**DOI:** 10.1371/journal.pone.0126647

**Published:** 2015-05-19

**Authors:** Patumrat Sripan, Sophie Le Coeur, Billy Amzal, Lily Ingsrisawang, Patrinee Traisathit, Nicole Ngo-Giang-Huong, Kenneth McIntosh, Tim R. Cressey, Suraphan Sangsawang, Boonsong Rawangban, Prateep Kanjanavikai, Jean-Marc Tréluyer, Gonzague Jourdain, Marc Lallemant, Saïk Urien

**Affiliations:** 1 Department of Statistics, Kasetsart University, Bangkok, Thailand; 2 Institut de recherche pour le développement (IRD) UMI 174-PHPT, Marseille, France; 3 Ecole Doctorale de Santé Publique, Université Paris-Sud, Paris, France; 4 Department of Medical Technology, Chiang Mai University, Chiang Mai, Thailand; 5 Harvard School of Public Health, Boston, MA, United States of America; 6 Institut d'Etudes Démographiques, Paris, France; 7 LASER Analytica, London, United Kingdom; 8 Department of Statistics, Chiang Mai University, Chiang Mai, Thailand; 9 Boston Children's Hospital, Boston, MA, United States of America; 10 Department of Pediatrics, Harvard Medical School, Boston, MA, United States of America; 11 Health Promotion Center Region 10, Chiang Mai, Thailand; 12 Nopparat Rajathanee Hospital, Bangkok, Thailand; 13 Banglamung Hospital, Chonburi, Thailand; 14 EAU08 Université Paris Descartes, Sorbonne Paris Cité, Paris, France; 15 Unité de Recherche Clinique, AP-HP, Hôpital Tarnier, Paris, France; 16 CIC1419 INSERM, Cochin-Necker, Paris, France; University of Washington, UNITED STATES

## Abstract

**Background:**

Antiretroviral treatments decrease HIV mother-to-child transmission through pre/post exposure prophylaxis and reduction of maternal viral load. We modeled *in-utero* and *intra-partum* HIV transmissions to investigate the preventive role of various antiretroviral treatments interventions.

**Methods:**

We analysed data from 3,759 women-infant pairs enrolled in 3 randomized clinical trials evaluating (1) zidovudine monotherapy, (2) zidovudine plus perinatal single-dose nevirapine or (3) zidovudine plus lopinavir/ritonavir for the prevention of mother-to-child transmission of HIV in Thailand. All infants were formula-fed. Non-linear mixed effect modeling was used to express the viral load evolution under antiretroviral treatments and the probability of transmission.

**Results:**

Median viral load was 4 log_10_ copies/mL (Interquartile range: 3.36–4.56) before antiretroviral treatments initiation. An Emax model described the viral load time-course during pregnancy. Half of the maximum effect of zidovudine (28% decrease) and lopinavir/ritonavir (72% decrease) were achieved after 98 and 12 days, respectively. Adjusted on viral load at baseline (Odds ratio = 1.50 [95% confidence interval: 1.34, 1.68] per log_10_ copies/mL increment), antiretroviral treatments duration (OR = 0.80 [0.75, 0.84] per week increment) but not the nature of antiretroviral treatments were associated with *in-utero* transmission. Adjusted on gestational age at delivery (<37 weeks, OR = 2.37 [1.37, 4.10]), baseline CD4 (Odds ratio = 0.79 [0.72, 0.88] per 100 cells/mm3 increment) and predicted viral load at delivery (OR = 1.47 [1.25, 1.64] per log_10_ copies/mL increment), single-dose nevirapine considerably reduced *intra-partum* transmission (OR = 0.32 [0.2, 0.51]).

**Conclusion:**

These models determined the respective contributions of various antiretroviral strategies on prevention of mother-to-child transmission. This can help predict the efficacy of new antiretroviral treatments and/or prevention of mother-to-child transmission strategies particularly for women with no or late antenatal care who are at high risk of transmitting HIV to their offspring.

**Trial Registration:**

This analysis is based on secondary data obtained from three clinical trials. ClinicalTrials.gov. NCT00386230, NCT00398684, NCT00409591.

## Introduction

Mother-to-child transmission (MTCT) of HIV can occur during pregnancy (*in-utero*), labor/delivery (*intra-partum*) or breastfeeding (*post-partum*). In the absence of antiretroviral treatment (ART), the transmission rate is 10% during pregnancy, 15% during labor and delivery and 10% during breastfeeding [1]. Viral load (VL) is the main predictor of MTCT [2]. Over the last two decades, studies have demonstrated that antiretroviral treatment during gestation, *intra-partum*, and breastfeeding dramatically reduces MTCT [3–6]. ART reduces MTCT through two complementary mechanisms: ART can reduce viral load and decrease the infant exposure to maternal viruses. Antiretrovirals can also cross the placenta and provide pre/post-exposure prophylaxis to the fetus and infant [7,8]. The 2013 World Health Organization (WHO) consolidated guidelines on the use of antiretroviral drugs for treating and preventing HIV infection [9] recommend that all HIV infected pregnant and breastfeeding women should initiate antiretroviral therapy as early as possible in pregnancy and maintain it at least for as long as the child is exposed to HIV.

Due to the high efficacy of current strategies leading to transmission rates as low as 2% or less, the evaluation of new drugs or drug combinations or new strategies for prevention of mother to child transmission (PMTCT) of HIV requires very large sample sizes to demonstrate efficacy improvements or non-inferiority. Modelling the efficacy of ARTs on MTCT, taking into account known risk factors, becomes increasingly important to gain prior information and optimize clinical trial design.

The objective of this work was to model *in-utero* and *intra-partum* HIV transmissions and, after adjusting for known risk factors, to investigate the role of various antiretroviral drug interventions for the PMTCT.

## Material and Methods

### Patients

Data were collected from pregnant women and infants who participated in three perinatal HIV prevention trials in Thailand.

#### PHPT-1

(NCT00386230)[4], carried out between 1996 and 2000, was a randomized, double-blind equivalence trial which compared the efficacy of zidovudine (ZDV) starting at 28 weeks' gestation plus 6 weeks of treatment in infants (the reference, “long-long” regimen) versus zidovudine starting at 35 weeks' gestation, with 3 days of zidovudine in infants (“short-short” regimen), and long-short and short-long regimens.

#### PHPT-2

(NCT00398684) [10], carried out between 2000 and 2004, was a randomized, double-blind trial of three treatment regimens, which evaluated the efficacy of single dose nevirapine (sdNVP) in mother during labor and in neonates or in mother only in addition to zidovudine during the third trimester of pregnancy and at least one week in children. Women enrolled in two PHPT-2 sub-studies (i) an open-label study for women who presented after 28 weeks gestation and (ii) a nevirapine pharmacokinetic study[11] were also included.

#### PHPT-5 first phase

(NCT00409591) [12], carried out between 2008 and 2010, was a randomized, 3-arm, double-blind trial. The three ARV strategies initiated during the third trimester were (i) maternal ZDV plus sdNVP at onset of labour and two infant NVP doses (at birth and 48 hours of life), (ii) maternal ZDV and two infant NVP doses, (iii) Maternal ZDV plus lopinavir/ritonavir (LPV/r), with no maternal or infant NVP.

CD4 cell count and viral load were performed before starting antiretrovirals and during pregnancy. Viral loads were repeated at variable times during pregnancy and at delivery. All infants were formula fed. Of the 3,948 confirmed HIV positive pregnant women, 71 were lost to follow up, withdrew consent or died before delivery. Therefore, 3,877 women delivered at the PHPT hospital sites. After exclusion of 28 mothers of a stillborn child, 73 women who had no VL evaluation and 17 women who were receiving HAART for their own health, the aggregated dataset included 3,759 women with at least one VL sample during pregnancy.

### Maternal plasma HIV-1 RNA levels

In all studies the maternal HIV-RNA measurement was planned prior to antiretroviral prophylaxis/treatment initiation to assess risk factors of transmission. In PHPT-1 and PHPT-2, VL samples were primarily collected for measurements at entry and delivery, while for PHPT-5, VL was measured monthly to assess HIV-RNA kinetics on antiretroviral drugs.

Plasma VL was assessed at the central PHPT laboratory in Chiang Mai University. Samples from PHPT-1 and PHPT-2 studies were tested using Cobas Amplicor HIV-1 Monitor kit version 1.5 (Roche Molecular Systems, USA) with a limit of quantification of 400 copies/mL; and samples from PHPT-5 first phase using the Abbott m2000 RealTime© HIV-1 assay (limit of quantification 40 copies/mL).

### HIV status in infants and timing of transmission

To determine HIV infection status in infants, peripheral blood was drawn and spotted onto filter papers, dried and stored at -20°C before shipment to a central laboratory. Each of the PHPT samples were collected at birth, 6 weeks, 4 and 6 months. PHPT-5 samples were collected at birth, 7–10 days, 1, 2, 4 and 6 months of age.

In the original trials, infants were considered confirmed HIV-infected if samples obtained on two separate occasions were found positive by HIV-1 DNA PCR and confirmed HIV-uninfected if samples obtained on two separate occasions after one month of age were negative [13]. When only one DNA PCR was available and positive, infants were considered unconfirmed HIV-infected. When only one DNA PCR was available after the 1^st^ week of life and negative, infants were considered unconfirmed HIV-uninfected. When only one DNA PCR was available within the 1^st^ week of life and negative, infants were considered as indeterminate [13]. In the present analysis, unconfirmed HIV infected infants were considered HIV-infected and unconfirmed HIV-uninfected infants were considered HIV-uninfected, while indeterminate infants were excluded.

Infants with a positive DNA PCR result during the first week of life were considered to be infected during pregnancy (“*in-utero*” transmission); infants with negative HIV-DNA PCR results during the first week of life but with a subsequent positive result were considered to be infected during labor or delivery (“*intra-partum*” transmission)[13]. Twins were considered a single entity and discordant twins were counted as one infected infant.

### Ethics

Each of the PHPT perinatal study protocols and their amendments, as well as the use of data for this analysis received ethical clearance from the Thai Ministry of Public Health, the Harvard School of Public Health and Chiang Mai University Faculty of Medical Associated Sciences Ethics Committees. The consent procedures were reviewed and approved by the ethics committees. Before enrollment, all women provided written informed consent for their participation and that of their infants.

### Modeling of viral load time course during pregnancy

An Emax model was chosen since it is based upon pharmacological principles, i.e. the theory of drug action mediated by ligand-receptor interaction which translates in an hyperbolic equation. Because ZDV and LPV/r have 2 distinct sites and mechanisms of action, the effects were considered to be additive [14]. A proportional effect Emax model [14] was applied to describe the viral load at time *T* during pregnancy (VL, expressed in log values). The model took into account VL before treatment initiation (VL_0_) and the duration and nature of the 2 ARTs including ZDV and LPV/r (S1 Dataset) was composed as follows.

$$VL_{T}=VL_{0}\times \left(1-\sum_{j=1}^{2}\frac{E_{MAX,j}\times T_{{}_{j}}^{\gamma_{j}}}{T_{{}_{50,j}}^{\gamma_{j}}+T_{{}_{j}}^{\gamma_{j}}}\right)$$

Model parameters were

*VL*_0_: VL before treatment

*E*_*MAX*_: Treatment maximum effect

γ: Hill coefficient for treatment effect

*T*_50_: Treatment duration to reach half of *E*_*MAX*_

Since ZDV and LPV/r have distinct sites and mechanisms of action, the effects of ZDV and LPV/r administered in combination were assumed to be additive [14]. Because the inhibition cannot exceed 100%, *E*_*MAX*,*LPV*_ was deduced from *E*_*MAX*,*ZDV*_ by

$$E_{MAX,LPV}=1-E_{MAX,ZDV}$$

Where

*E*_*MAX*,*ZDV*_: ZDV maximum effect

*E*_*MAX*,*LPV*_: LPV/r maximum effect

Interindividual variability was modeled using an exponential error model, with *η*_*i*_ being the interindividual random effect with mean 0 and variance *ω* ^2^.

$$E_{MAX,i}=E_{MAX,pop}\times \exp(\eta_{i,E\max})\;\text{with}\;\eta_{i,E\max}\sim N\left(0,\omega_{E\max}^{2}\right)$$

$$\gamma_{i}=\gamma_{,pop}\times \exp(\eta_{i,\gamma })\;\text{with}\;\eta_{i,\gamma }\sim N\left(0,\omega_{\gamma }^{2}\right)$$

$$T_{50,}{}_{i}=T_{50}{}_{,pop}\times \exp(\eta_{i,T_{50}})\;\text{with}\;\eta_{i,T50}\sim N\left(0,\omega_{T50}^{2}\right)$$

### Modeling of *in-utero* and *intra-partum* transmissions

*In-utero* and *intra-partum* transmissions were treated as independent outcomes. Logistic regression models with random effect (*η*) were developed to predict *in-utero* and *intra-partum* transmission according to relevant risk factors. For each odds ratio, point estimate and 95% confidence intervals are provided.

We investigated known risk factors for mother-to-child transmission of HIV [2,15,16]. *In-utero* transmission was assumed to depend on VL_0_ (log_10_copies/mL), drug(s) treatment duration(s) in weeks, CD4 count before treatment (CD4_BASELINE_) and gestational age (GA) at treatment initiation and at delivery (S2 Dataset). The risk of *intra-partum* transmission was dependent on VL at delivery (VL_DELIVERY_), itself predicted by the VL time-course model, perinatal NVP (sdNVP administered at onset of labor or during the first hours of life or both), maternal ZDV loading dose during labor, delivery mode, premature labor (Gestational age (GA) <37 weeks) and ART(s) administered to infants during the first weeks of life (S3 dataset).

### Data analysis

VL time-courses and MTCT events were analysed using a non-linear mixed effect modeling approach. Parameters were estimated by computing the maximum likelihood estimators without any approximation of the model (no linearization) using the stochastic approximation expectation maximization (SAEM) algorithm combined to a Markov Chain Monte Carlo (MCMC) procedure. Data were analysed using MONOLIX (version 4.1.2, http://www.lixoft.com/) [17,18]. VL counts were log transformed and residual variability was described by an additive error, whereas an exponential model was used for between-subject variabilities (*η*). Data below the limit of quantification were left-censored [19]. The effect of a covariate on a structural parameter was retained if it produced a decrease in the Bayesian Information Criterion (BIC) compared to the baseline model i.e. the covariate-free model. A smaller BIC value signifies a model that better fits the data [20].

The logistic model for MTCT events analysis was written in a MLXTRAN script file (S1 MLXTRAN scripts); the random effect *η* was assumed to be normally distributed. In the univariate analyses, variables that both decreased BIC and had acceptable relative standard error (RSE<50%) were considered as significant factors to be included in the multivariable analyses. In the multivariable analysis, these variables were added one by one considering the largest drops in the BIC value to define the final model.

### Visual predictive check (VPC) evaluation

Simulated VL time-courses were compared with the observed data to evaluate the performance of the model. The vector of model parameters from 400 replicates of the database was simulated. The 5^th^, 50^th^ and 95^th^ percentiles of the simulated dependent variables at each time were then overlaid on the observed data. The proportion of observed MTCT with their confidence intervals were plotted as a function of significant predictors. The 5^th^, 50^th^ and 95^th^ percentiles of the model predictions were simultaneously plotted. Visual inspection was used to confirm that the observed proportions were included in the limits defined by the percentiles curves. The residual sum of square (RSS) was provided in addition to graphical check.

## Results

### Characteristics of the study population

**Fig 1:**
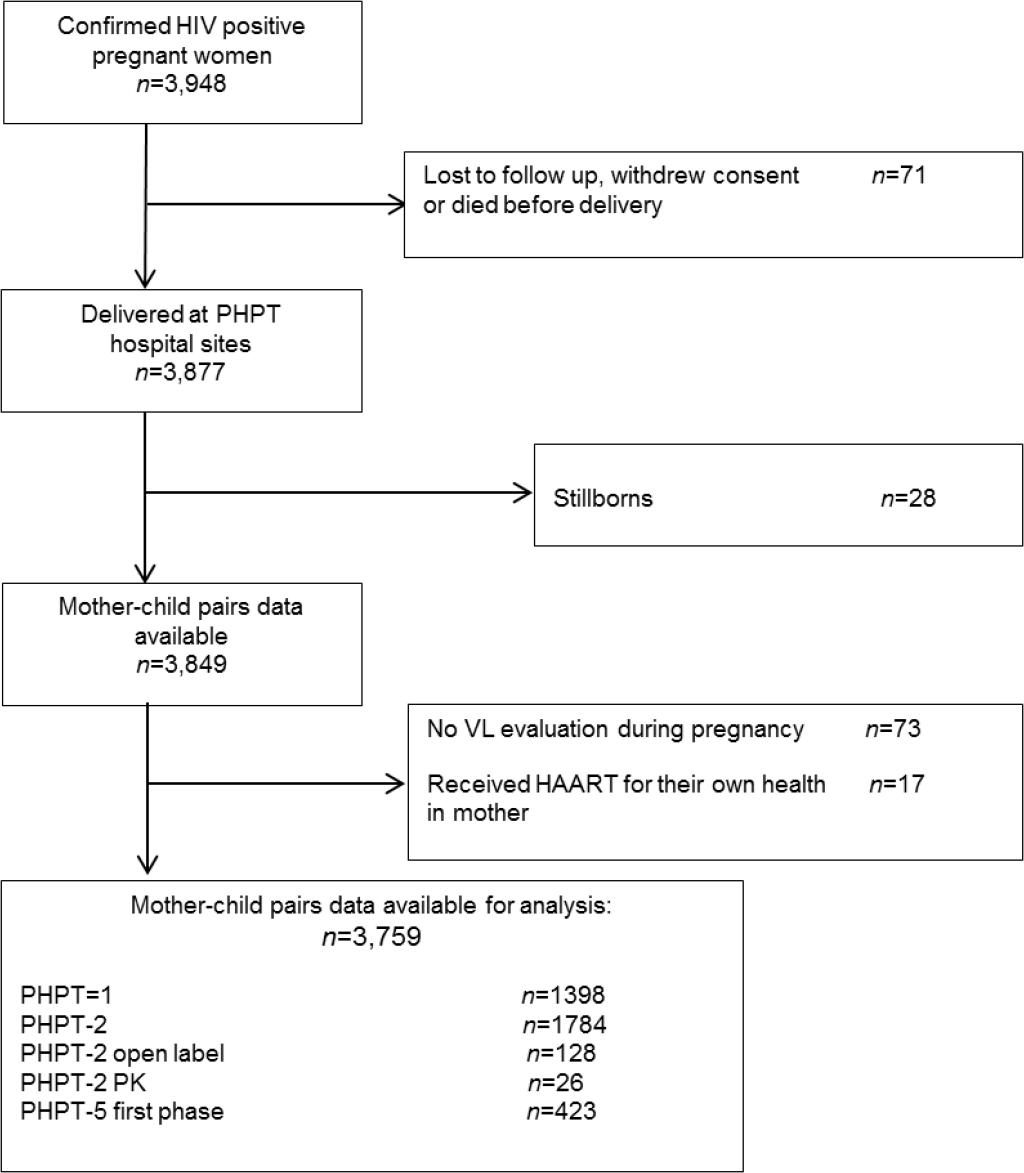
Population disposition.

A total of 3,759 HIV-infected pregnant women enrolled in the PMTCT studies from 1996 to 2010 were included (Fig 1). Table 1 presents the baseline and delivery characteristics of the women included in the analysis and the treatments they received.

**Table 1:** Characteristics of pregnant women and ARTs for perinatal HIV prevention according to study.

Variable	PHPT-1	PHPT-2	PHPT-2 OPEN Label	PHPT-2 PK	PHPT-5	Total
Number of women with at least 1 VL sample	1,398	1,784	128	26	423	3,759
**Characteristics of pregnant women**						
Gestational age at enrollment (weeks)						
Median	28.0	31.0	38.4	33.9	29.0	29.6
Interquartile range	27.7 to 28.2	30.1 to 33.1	36.5 to 40.0	31.3 to 37.3	28.0 to 30.0	28.0 to 31.6
CD4 at enrollment (cell count/ mm^3^)						
Median	360	376	368	454	458	385
Interquartile range	240 to 500	247 to 528	243 to 542	270 to 582	368 to 576	260 to 526
Gestational age at delivery (weeks)						
Median	39.0	38.6	38.6	39.4	38.7	38.7
Interquartile range	38.0 to 40.0	37.9 to 39.6	37.0 to 40.0	38.1 to 40.6	37.8 to 39.7	37.9 to 39.7
Type of delivery						
1) emergency C/section	141 (10%)	265 (15%)	21 (16%)	7 (27%)	0 (0%)	434 (11%)
2) planned C/section	111 (8%)	105 (6%)	9 (7%)	3 (11%)	59 (14%)	287 (8%)
3) vaginal delivery	1,146 (82%)	1,414 (79%)	98 (77%)	16 (62%)	364 (86%)	3,038 (81%)
**Maternal treatment**						
ZDV during pregnancy	1,387 (>99%)	1,784 (100%)	73 (57%)	23 (88%)	423 (100%)	3,690 (98%)
ZDV duration (days)						
Median	57	67	0	52	71	65
Interquartile range	30 to 79	52 to 77	0 to 11.5	30 to 61	60 to 78	39 to 77
LPV during pregnancy	-	-	-	-	145 (4%)	145 (4%)
LPV duration (days)						
Median	-	-	-	-	69	69
Interquartile range	-	-	-	-	55 to 77	55 to 77
NVP at onset of labor	-	1407 (79%)	110 (86%)	26 (100%)	133 (31%)	1,676 (45%)
ZDV loading dose at onset of labor	1,336 (96%)	1,777 (>99%)	119 (93%)	26 (100%)	410 (97%)	3,668 (98%)
**Infant treatment**						
ZDV prophylaxis	1,381 (99%)	1,779 (>99%)	127 (99%)	26 (100%)	420 (99%)	3,733 (99%)
ZDV duration (days)						
Median	41	11	44	7	7	11
Interquartile range	3 to 42	10 to 14	41 to 48	6 to 7	7 to 7	7 to 41
Postnatal NVP (infants)	-	729 (41%)	134 (97%)	26 (100%)	275 (65%)	1,154 (31%)
Perinatal NVP^a^	-	1,424 (80%)	126 (98%)	26 (100%)	276^b^ (65%)	1,852 (49%)

^a^ sdNVP either in the woman at onset of labor, in the infant or in both

^b^ only in women who did not receive LPV/r

The HIV status of the infants were as follows: 174 (5%) confirmed HIV-infected, 3,411 (91%) confirmed HIV-uninfected, 9 (<1%) unconfirmed HIV-infected, 113 (3%) unconfirmed HIV-uninfected and 52 (1%) indeterminate. According to the definition for this analysis, there were 183 HIV transmissions and 52 indeterminate infants were excluded from the transmission analysis. Among the infected infants, there were 80 *in-utero* and 103 *intra-partum* transmissions.

### ARTs during pregnancy

Of the 3,759 mother-infant pairs analysed, 1,751 (47%) received mother-infant ZDV monotherapy and 1,851 (49%) mother-infant ZDV plus perinatal sdNVP. In addition 145 (4%) mothers received ZDV plus LPV/r during the third trimester, without perinatal sdNVP.

### VL time-course modeling

**Fig 2:**
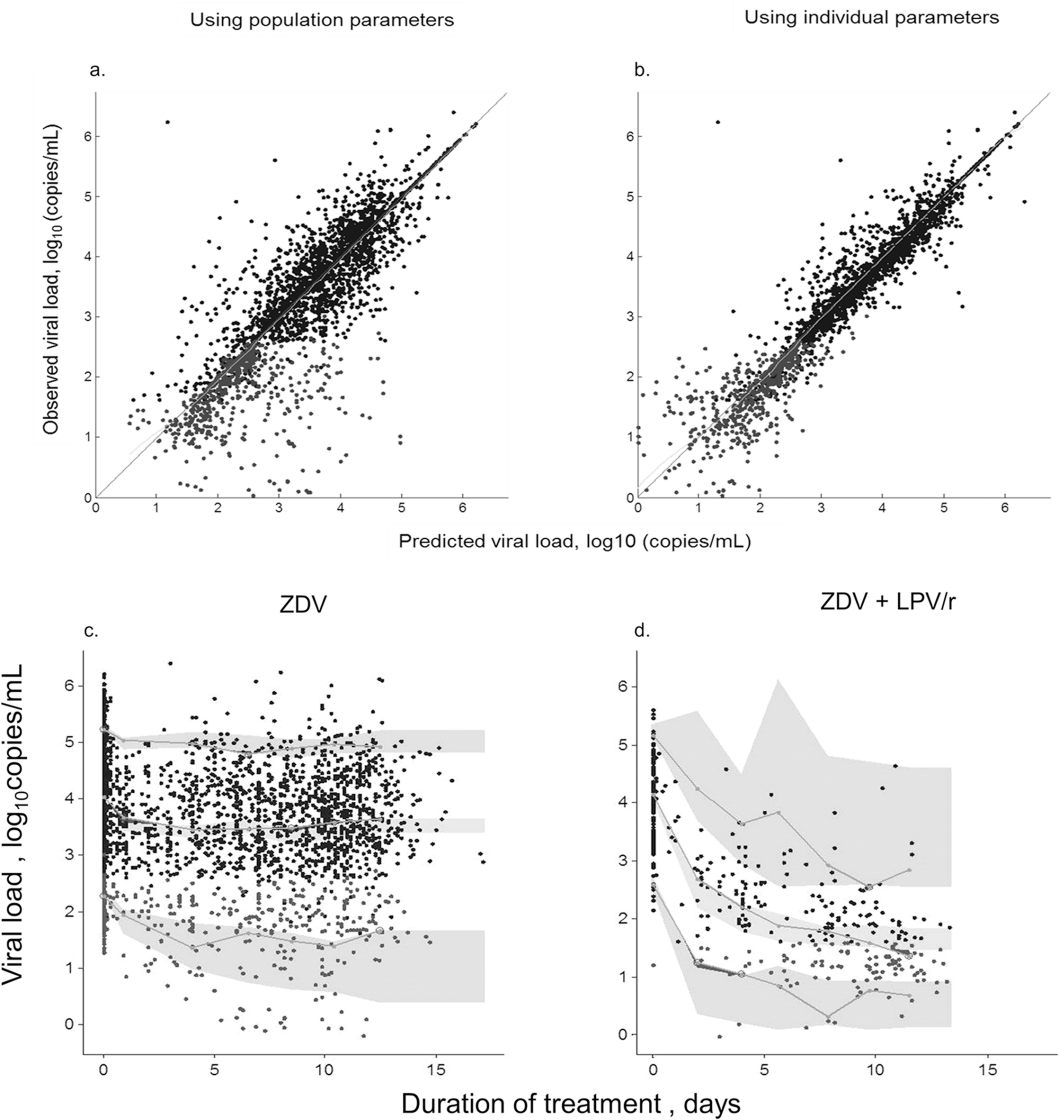
Diagnostic plots for viral load time-course model. Top: 2a and 2b: Observed versus model predicted viral load values (expressed as log_10_ copies) of the population and individual predictions respectively. Solid black circles, measure values; grey symbols, simulation of below the limit of quantification data. Line, identity line. Bottom: Visual predictive check plots. (2c) Women receiving only zidovudine (ZDV); (2d) women receiving zidovudine plus lopinavir/ritonavir (ZDV+LPV/r).The lines denote the median, 5^th^ and 95^th^ percentiles for the observed data. The grey areas stand for the 95% confidence intervals of the median, 5^th^ and 95^th^ model prediction percentiles.

A total of 5,576 VL measurements in 3,759 subjects were available for modeling (median 1 measurement per patient, range 1 to 6). Sixty five percent of the women had only 1 measurement (all but 5% of these at ART initiation), and 35% had at ≥2 measurements. Median VL was 4 log_10_ copies/mL (IQR: 3.36–4.56) before ART initiation and 3.51 log_10_copies/ mL (IQR: 2.89–3.34) at delivery. VL at any time point during pregnancy was dependent on baseline VL and ZDV and LPV/r treatment durations and was well described by a combined Emax model. The Hill coefficient for ZDV effect (γ_ZDV_) *γ*_*ZDV*_ was close to 1 and thus was fixed to 1. The *η* parameters for γ_ZDV_, LPV/r duration to reach half of *E*_*MAX*,*LPV*_ (*T*_50,*LPV*_) and the Hill coefficient for LPV/r (γ_*LPV*_) *γ*_*ZDV*_ were not statistically significant. Removing them from the model did not alter the quality of the fit or further decreased the BIC value (final model, BIC = 2113.33). None of the other covariates, including CD4 cell count and GA at baseline, had a significant effect on model parameters. All parameters were well estimated with RSE below 30% (Table 2). The model estimated that half of the maximum effect of ZDV (28% VL decrease from baseline) and LPV/r (72% decrease) were observed after 98 and 12 days respectively. Using the population parameter, the RSS was 747.39 while it was 369.20 when using individual parameter. The observed vs. model-predicted plots are shown in Fig 2A and 2B (top). The visual predictive checks are shown in Fig 2C and 2D (bottom).

**Table 2:** Population parameter estimates of HIV time-course model for 3,759 HIV-1-infected mothers enrolled in the PHPT-1, PHPT-2, and PHPT-5 studies.

Parameters	Estimate	SE^a^	RSE^b^ (%)
*Structural model*			
*T*_50,*ZDV*_	98.3	12	12
γ_ZDV_	1 (fixed)	-	-
*T*_50,*LPV*_	11.6	3.3	29
γ_LPV_	0.28	0.049	18
*E*_*MAX*,*ZDV*_	0.285	0.016	6
*E*_*MAX*,*LPV*_	0.715	-	-
*Statistical model*			
$\omega_{\eta_{T50,zdv}}$	2.34	0.15	6
$\omega_{\eta_{Emax,zdv}}$	0.852	0.043	5
*σ*_*VL*_ ^c^	0.197	0.0028	1

^a^ SE, standard error of estimate

^b^ RSE%, relative standard error (standard error of estimate / estimate*100)

^c^ *σ*_*VL*_, residual (square roots of variances)

### MTCT modeling

The MTCT models were built step by step from the basic model (without explanatory variable). Viral load at delivery (VL_DELIVERY_) was estimated through the VL time course final model using individual parameters.

*In-utero* transmission. Upon univariate analysis, CD4 count, gestational age, VL before treatment initiation and ZDV duration caused a drop in the BIC, indicating significant effects of these variables. In the multivariable analysis, only ZDV duration and VL before treatment initiation remained independently associated with *in-utero* transmission (Table 3). LPV/r duration and baseline CD4 count were not significantly associated with *in-utero* transmission.

**Table 3:** The univariate and multivariable analyses of the HIV *in-utero* model using data from 3,707 HIV-1-infected mothers enrolled in the PHPT-1, PHPT-2, and PHPT-5 studies.

Predictors	Logit coefficient(95%CI)	Odds ratio(95%CI)	RSE (%)^a^	BIC^b^
Univariate analysis				
Baseline model	-3.71 (-3.89, -3.53)	-	2	791.42
ZDV duration (weeks)	-0.09 (-0.15, -0.04)	0.91 (0.86, 0.96)	29	772.04
VL before treatment initiation (log_10_ copies/mL)	0.23 (0.158, 0.31)	1.26 (1.17, 1.36)	17	778.06
CD4 (per 100 cell count)	-0.13 (-0.22, -0.04)	0.88 (0.80, 0.96)	34	784.04
Gestational age at baseline (days)	0.003 (0.001, 0.005)	1.003 (1.001, 1.005)	33	794.26
Multivariable analysis				
Model 1				740.13
VL	0.43 (0.29, 0.58)	1.54 (1.34, 1.78)	17	
ZDV duration	-0.21 (-0.27, -0.15)	0.81 (0.77, 0.86)	15	
CD4	-0.19 (-0.30, -0.08)	0.83 (0.74, 0.92)	30	
Model 2				746.69
VL before treatment	0.44 (0.22, 0.66)	1.55 (1.25, 1.93)	26	
ZDV duration	-0.22 (-0.28, -0.16)	0.80 (0.76, 0.85)	13	
GA at baseline	3.44e-007 (-0.004, 0.004)	1.00 (0.99, 1.004)	6.25e+005	
Final model^c^				735.96
VL before treatment	0.41 (0.30, 0.52)	1.50 (1.34, 1.68)	14	
Duration of ZDV	-0.23 (-0.28,-0.17)	0.80 (0.75, 0.84)	13	

^a^ RSE%, relative standard error (standard error of estimate / estimate*100)

^b^ Bayesian Information Criterion

^c^ Random effect of individuals: *η*~*N*(0,0.527^2^)

The final model was:

$$\text{Logit}(\text{transmission})=-3.91+0.41\times {\text{VL}}_{0}\;-\;0.23\times {\text{ZDV}}_{\text{DURATION}}$$

where ZDV_DURATION_ is the ZDV duration during pregnancy (weeks).

**Fig 3:**
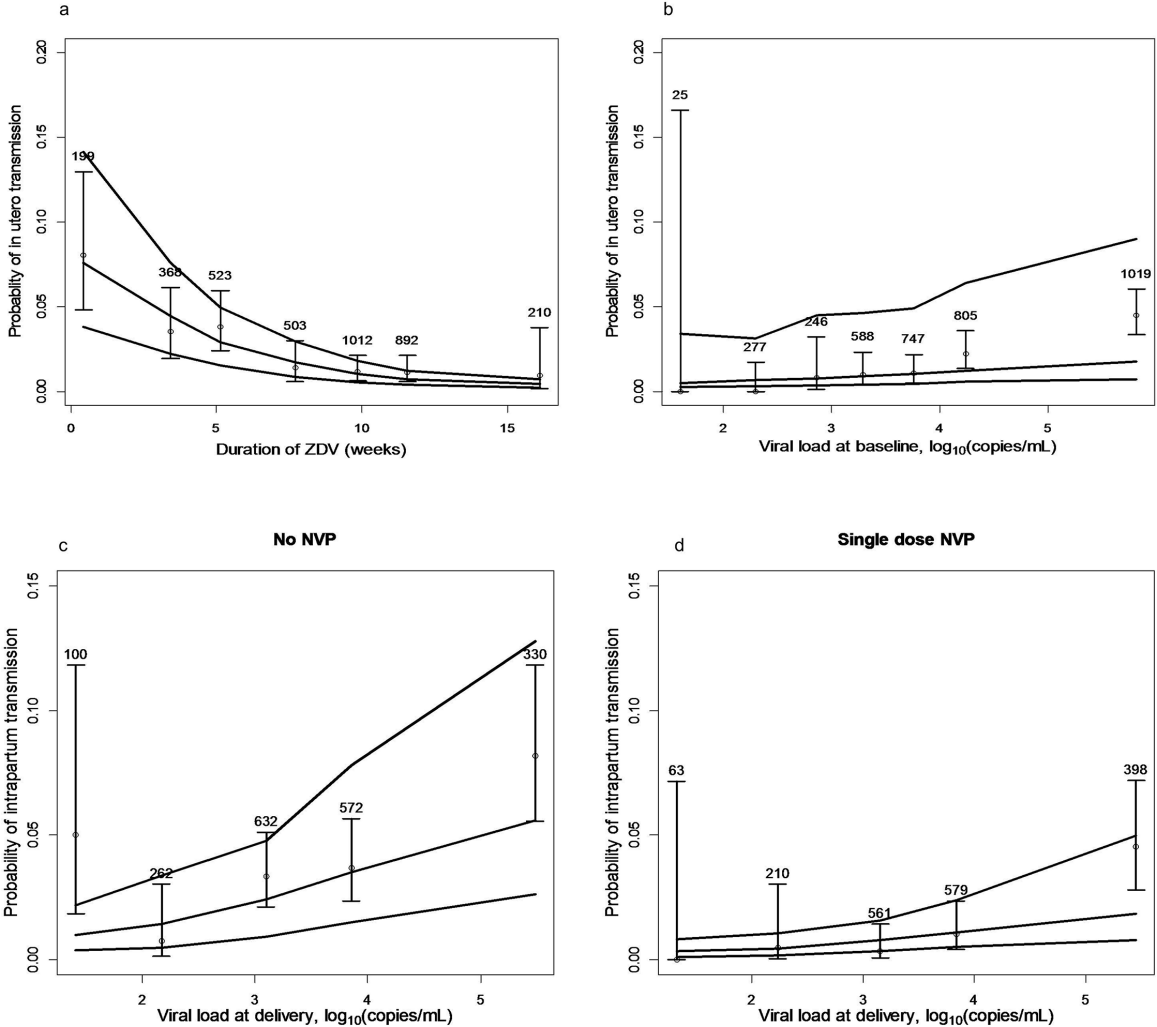
Transmission probabilities according to zidovudine duration, viral load at baseline/delivery, and nevirapine intake. A. Probability of *in-utero* HIV transmission as a function of zidovudine duration; B. Probability of *in-utero* HIV transmission as a function of viral load at baseline. The lines denote the median, 5^th^ and 95^th^percentiles of the model predictions. The open circles stand for the observed mean proportion of transmission, the solid vertical segments denote the corresponding 95% confidence intervals (numbers at top of each segment stand for the number of women in each time interval or VL interval). C. Probability of *intra-partum* HIV mother-to-child transmission as a function of viral load at delivery without single dose nevirapine; D. Probability of *intra-partum* HIV mother-to-child transmission as a function of viral load at delivery with single dose nevirapine.

The probability of *in-utero* HIV transmission as a function of zidovudine duration is shown in Fig 3A, and that of *in-utero* HIV transmission as a function of viral load at baseline in Fig 3B.

*Intra-partum* transmission. Perinatal sdNVP, duration of ZDV, premature labor (GA at delivery<37 weeks)viral load at delivery and CD4 cell count were significantly associated with *intra-partum* transmission in the univariate analyses, while mode of delivery, ZDV loading dose and infants ZDV prophylaxis were not.

In the multivariable analysis, final model included VL at delivery, perinatal sdNVP administration, CD4 cell count and premature labor (Table 4). The duration of ZDV was no longer significant when other factors were included.

**Table 4:** The univariate and multivariable analyses of the HIV intra-partum transmission model using data from 3,707 HIV-1-infected mothers enrolled in the PHPT-1, PHPT-2, and PHPT-5 studies.

Predictors	Logit coefficient(95%CI)	Odds ratio(95%CI)	RSE (%)^a^	BIC^b^
Univariate analysis				
Baseline model	-3.29 (-3.43, -3.15)	-	2	968.77
ZDV duration (weeks)	-0.049 (-0.09, -0.01)	0.95 (0.91, 0.99)	46	956.07
VL at delivery (log_10_ copies/mL)	0.17 (0.08, 0.26)	1.18 (1.08, 1.30)	26	953.04
CD4 (per 100 cell count)	-0.18 (-0.27, -0.09)	0.83 (0.77, 0.91)	25	942.79
Premature labor	0.81 (0.30,1.32)	2.25 (1.35, 3.75)	32	957.50
Perinatal NVP	-1.10 (-1.53, -0.67)	0.33 (0.22, 0.51)	20	943.94
Multivariable analysis				
Model 1				926.61
Perinatal NVP	-0.97 (-1.44, -0.50)	0.38 (0.24, 0.61)	25	
CD4	-0.22 (-0.33, -0.12)	0.80 (0.72, 0.88)	23	
Model 2				916.58
Perinatal NVP	-1.13 (-1.58, -0.68)	0.32 (0.21, 0.51)	21	
CD4	-0.23 (-0.33, -0.12)	0.80 (0.72, 0.88)	16	
VL at delivery	0.42 (0.29, 0.55)	1.52 (1.34, 1.74)	23	
Model 3				922.80
Perinatal NVP	-1.15 (-1.62, -0.68)	0.32 (0.20, 0.51)	21	
CD4	-0.153 (-0.25, -0.05)	0.86 (0.78, 0.95)	33	
VL at delivery	0.634 (0.51, 0.76)	1.88 (1.67, 2.13)	10	
ZDV duration	0.0001 (-0.0072, 0.0073)	1.00 (0.99, 1.01))	3.67e+004	
Final model^c^				916.61
Perinatal NVP	-1.13 (-1.58, -0.68)	0.32 (0.21, 0.51)	21	
CD4	-0.23 (-0.34, -0.13)	0.79 (0.72, 0.88)	22	
VL at delivery	0.36 (0.23, 0.50)	1.44 (1.26, 1.64)	19	
Premature labor	0.86 (0.31, 1.41)	2.37 (1.37, 4.10)	32	

^a^RSE%, relative standard error (standard error of estimate / estimate*100)

^b^ Bayesian Information Criterion

^c^ Random effect of individuals: *η*~*N*(0,0.73^2^)

The final model was:

$$\text{Logit}\left(\text{transmission}\right)=\text{-}3.96+0.36{\text{xVL}}_{\text{DELIVERY}}\;\text{-}\;1.13\text{xsdNVP}+0.86\text{xPremature}\;\text{-}0.23{\text{xCD4}}_{\text{BASELINE}}$$

where 1 CD4_BASELINE_ unit is 100 cells /mm^3^, NVP and GA_DELIVERY_ are binary (YES = 1 or NO = 0)

Fig 3C and 3D show the probability of *intra-partum* HIV transmission as a function of viral load at delivery without and with single dose nevirapine, respectively.”

## Discussion

The VL time-course model during pregnancy developed as a function of the type of treatment administered, its duration and the VL level at baseline, provided a good prediction of the VL level at delivery. This predicted VL could be used in the MTCT models. VL at treatment initiation and treatment duration were the main determinants of *in-utero* transmission, regardless of the ARV regimens used. High VL at delivery, absence of perinatal sdNVP and premature labor were associated with *intra-partum* transmission.

As previously shown in PACTG 076 [21], although ZDV monotherapy had only a slight effect on maternal VL (only 0.43 Log decrease in this analysis), it was very effective in reducing *in-utero* transmission. This is consistent with the accepted concept that ZDV, which cross the placenta freely, exerts its prophylactic effect largely through pre-post exposure prophylaxis [21]. In the late 1990, it was hypothesized that *in-utero* transmission would occur late in pregnancy [22]. This justified for the launch of several short ZDV course trials in developing countries [23–25]. However, this was not confirmed by the PHPT-1 trial where the rates of *in-utero* transmission were 1.6 versus 5.1% with long and short ZDV treatments, respectively [4,15]. This supports the WHO PMTCT 2013 guidelines recommending ART initiation as early as possible during pregnancy [9]. Although ZDV plus LPV/r was much more effective than ZDV alone in reducing VL, adding LPV/r to ZDV did not decrease further *in-utero* transmission [26].

Although it had no effect on *in-utero* transmission, the addition of LPV/r, which has limited perfusion through the placenta, had as expected a major effect on the VL at delivery (mean reduction, 2.18 log_10_copies/mL), and thus a major effect on *intra-partum* transmission. More importantly, after adjusting for all factors associated with transmission, perinatal sdNVP, in the mother only, the mother and her infant, or the infant only, markedly reduced the risk of *intra-partum* transmission at all viral load levels (Fig 3C and 3D).

Modeling of VL during LPV/r plus ZDV treatment (Fig 2D) showed that with a treatment duration less than 8–10 weeks before delivery, VL at delivery remained detectable in a large proportion of women. Accordingly, when mothers start HAART late in pregnancy, it is advisable to intensify the ART regimen by providing sdNVP to both mother and infant and a brief course of combined ART to the newborn in order to reduce the probability of *intra-partum* transmission [27–28]. Interestingly, in the presence of perinatal sdNVP, *intra-partum* transmission was only weakly associated with VL at delivery, indicating the potent pre/post exposure prophylactic effect of this drug.

Prematurity has been found to be associated with perinatal transmission in several studies [15,16,29]. It has been debated whether prematurity was a consequence of *in-utero* transmission or if premature infants were more vulnerable to HIV infection [30]. The fact that in this study prematurity was associated with *intra-partum* but not with *in-utero* transmission supports a higher vulnerability of premature children.

Several studies reported that planned caesarean section (CS) [31,32] reduced the risk of *intra-partum* transmission in particular when VL at delivery is high but, in this study, the percentage of women with planned C-section was too low (8%) to assess this factor.

This study has several limitations. In our definition of HIV status of the infants, we considered as infected or uninfected, infants with unconfirmed HIV status and excluded those who were indeterminate. However, a sensitivity analysis restricted to infants with confirmed HIV status provided very close results (data not shown). Datasets from other trials with different antiretroviral prophylaxis regimens could have been incorporated into the model but this would have perhaps offset the advantages of using data from trials performed in the same setting by the same team. Also, all subjects were from Thailand although there is no indication that risks of transmission and intervention effectiveness differ across ethnic groups.

In conclusion, our models provide insights on the respective roles of pre-exposure prophylaxis and maternal viral load reduction in preventing mother-to-child transmission according to the preventive strategy. With the regimens considered in this analysis, while the preventive effect of ART during pregnancy was essentially driven by transplacental pre/post exposure prophylaxis, both viral load reduction by the time of delivery and infant prophylaxis were important in preventing *intra-partum* transmission. Given the high efficacy of current interventions, clinical trials to test the efficacy of new antiretrovirals or PMTCT strategies have become more and more difficult to implement. A Bayesian approach with data from previous clinical trials could reduce sample sizes and help optimize trial design.

## Supporting Information

S1 Dataset Data used for the final viral load time-course model based on the MLXTRAN scripts. (CSV)

S2 Dataset Data used for the final *in-utero* transmission model based on the MLXTRAN scripts. (CSV)

S3 Dataset Data used for the final *intra-partum* transmission model based on the MLXTRAN scripts. (CSV)

S1 File MLXTRAN scripts. (DOC)
