# Correction: Modeling of *In-Utero* and *Intra-Partum* Transmissions to Evaluate the Efficacy of Interventions for the Prevention of Perinatal HIV

**DOI:** 10.1371/journal.pone.0130917

**Published:** 2015-06-26

**Authors:** Patumrat Sripan, Sophie Le Coeur, Billy Amzal, Lily Ingsrisawang, Patrinee Traisathit, Nicole Ngo-Giang-Huong, Kenneth McIntosh, Tim R. Cressey, Suraphan Sangsawang, Boonsong Rawangban, Prateep Kanjanavikai, Jean-Marc Tréluyer, Gonzague Jourdain, Marc Lallemant, Saïk Urien

There are errors in the author affiliations. The affiliations should appear as shown here:

Patumrat Sripan^1,2,3^, Sophie Le Coeur^2,4,6,^, Billy Amzal^2,7^, Lily Ingsrisawang^1^, Patrinee Traisathit^8^, Nicole Ngo-Giang-Huong^2,4,5^, Kenneth McIntosh^9,10^, Tim R. Cressey^2,4,5^, Suraphan Sangsawang^11^, Boonsong Rawangban^12^, Prateep Kanjanavikai^13^, Jean-Marc Tréluyer^14,15,16,^, Gonzague Jourdain^2,4,5^, Marc Lallemant^2^ and Saïk Urien^14,15,16^

**1** Department of Statistics, Faculty of Science, Kasetsart University, Bangkok, Thailand, **2** Institut de recherche pour le développement (IRD) UMI 174-PHPT, Marseille, France, **3** Ecole Doctorale de Santé Publique, Université Paris-Sud, Paris, France, **4** Department of Medical Technology, Faculty of Associated Medical Science, Chiang Mai University, Chiang Mai, Thailand, **5** Harvard School of Public Health, Boston, MA, United States of America, **6** Institut d'Etudes Démographiques, Paris, France, **7** LASER Analytica, London, United Kingdom, **8** Department of Statistics, Faculty of Science, Chiang Mai University, Chiang Mai, Thailand, **9** Boston Children's Hospital, Boston, MA, United States of America, **10** Department of Pediatrics, Harvard Medical School, Boston, MA, United States of America, **11** Health Promotion Center Region 10, Chiang Mai, Thailand, **12** Nopparat Rajathanee Hospital, Bangkok, Thailand, **13** Banglamung Hospital, Chonburi, Thailand, **14** EAU08 Université Paris Descartes, Sorbonne Paris Cité, Paris, France, **15** Unité de Recherche Clinique, AP-HP, Hôpital Tarnier, Paris, France, **16** CIC1419 INSERM, Cochin-Necker, Paris, France.
