# Correction: Modeling of *In-Utero* and *Intra-Partum* Transmissions to Evaluate the Efficacy of Interventions for the Prevention of Perinatal HIV

**DOI:** 10.1371/journal.pone.0137368

**Published:** 2015-08-28

**Authors:** Patumrat Sripan, Sophie Le Coeur, Billy Amzal, Lily Ingsrisawang, Patrinee Traisathit, Nicole Ngo-Giang-Huong, Kenneth McIntosh, Tim R. Cressey, Suraphan Sangsawang, Boonsong Rawangban, Prateep Kanjanavikai, Jean-Marc Tréluyer, Gonzague Jourdain, Marc Lallemant, Saïk Urien

Fig 2 is incorrect. The duration of treatment should be in weeks instead of days. The authors have provided a corrected version here.

**Fig 2:**
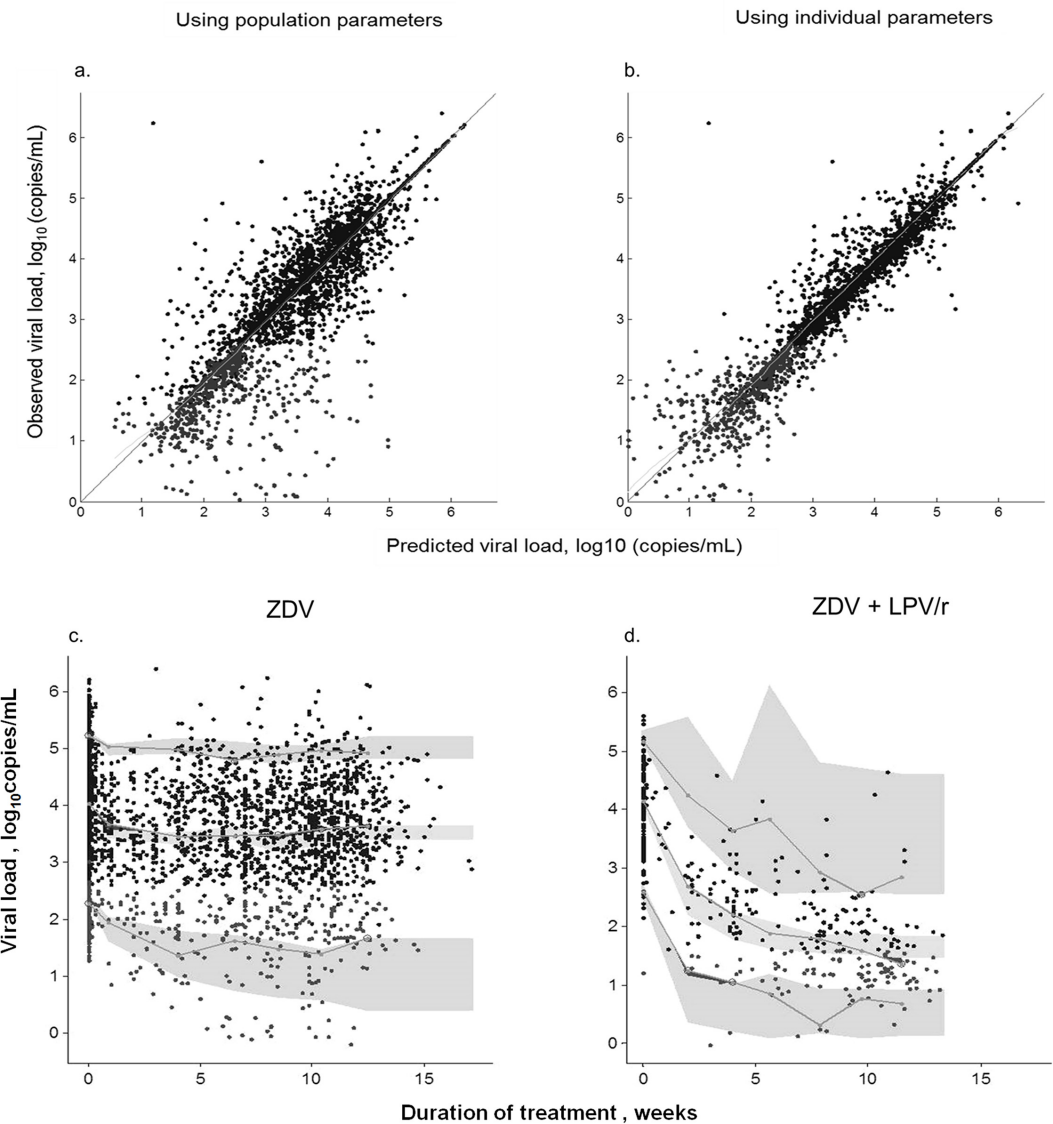
Diagnostic plots for viral load time-course model. Top: 2a and 2b: Observed versus model predicted viral load values (expressed as log_10_ copies) of the population and individual predictions respectively. Solid black circles, measure values; grey symbols, simulation of below the limit of quantification data. Line, identity line. Bottom: Visual predictive check plots. (2c) Women receiving only zidovudine (ZDV); (2d) women receiving zidovudine plus lopinavir/ritonavir (ZDV+LPV/r).The lines denote the median, 5^th^ and 95^th^ percentiles for the observed data. The grey areas stand for the 95% confidence intervals of the median, 5^th^ and 95^th^ model prediction percentiles.
