# Supplementary material for: Modeling of In-Utero and Intra-Partum Transmissions to Evaluate the Efficacy of Interventions for the Prevention of Perinatal HIV
Source: PLoS One. 2015 May 19;10(5):e0126647. doi: 10.1371/journal.pone.0126647 (PMC4438074; doi:10.1371/journal.pone.0126647)
Supplement: S1 File — (DOC) [file pone.0126647.s004.doc]

$PROBLEM VL time-course model

$PSI z50 lop50 glop emxz

; Hill coefficient for LPV/r effect

; emxz: maximum effect of ZDV

; z50: time to achieve half of maximum effect of ZDV

; lop50: time to achieve half of maximum effect of LPV/r

$REG vlinc tzdv tlopi durz durlp

; vlinc : VL before treatment initiation, Log copies/mL

; tzdv: duration of ZDV at VL measurement , days

; tlopi: duration of LPV/r at VL measurement , days

; durz: duration of ZDV during pregnancy , days

; durlp: duration of LPV/r during pregnancy , days

$EQUATION

if (tzdv>0) then

r1 = (z50/tzdv)

else

r1 = 0

end

if (r1 >0) then

effz = emxz/(1 + r1)

else

effz = 0

end

if (tlopi>0) then

r2 = (lop50/tlopi)**glop

else

r2 = 0

end

if (r2 >0) then

effl = (1 - emxz)/(1 + r2)

else

effl = 0

end

inh = 1 - effz - effl

vl = (vlinc)*inh ;; placebo hyperbol increase

effz2 = emxz*durz/(z50 + durz)

durlp2 = durlp**glop

effl2 = (1 - emxz)*durlp2/(durlp2 + lop50**glop)

vl2 = vlinc*(1 - effz2 - effl2)

$OUTPUT

OUTPUT1 = max(vl , 0)

$TABLE inh vl2

$PROBLEM in-utero transmission model

$PSI th0 th_vlinc th_durz

; th0: intercept

; th_vlinc: effect of VL before treatment initiation

; th_durz: effect of ZDV treatment

$REG vlinc durzw

; vlinc: VL before treatment initiation, Log copies/mL

; durzw: duration of ZDV, weeks

$CATEGORICAL(0,1)

LOGIT1(Y > 0) = th0 + th_vlinc*vlinc + th_durz*durzw

$OUTPUT

OUTPUT1 = LL1

$PROBLEM intrapartum transmission model

$PSI th0 th_nvp th_vldel th_cd4 th_gab

; th0: intercept

; th_nvp: effect of perinatal sdNVP

; th_vldel: effect of predicted VL at delivery

; th_cd4: effect of cd4

;th_gab: effect of premature labor(GA at delivery<37 weeks)

$REG nvp vl2saem gab cd4100

; nvp: perinatal sdNVP (yes/no)

;vl2saem: predicted at delivery, Log copies/mL

; gab: premature labor (yes/no)

;cd4100: CD4 , 100 cell count

$CATEGORICAL(0,1)

LOGIT1(Y >0) = th0 + th_nvp*nvp + th_vldel*vl2saem + th_cd4*cd4100 + th_gab*gab

$OUTPUT

OUTPUT1 = LL1
